# Physicians' Acceptance of Triage Guidelines in the Context of the COVID-19 Pandemic: A Qualitative Study

**DOI:** 10.3389/fpubh.2021.695231

**Published:** 2021-07-30

**Authors:** Federica Merlo, Mattia Lepori, Roberto Malacrida, Emiliano Albanese, Marta Fadda

**Affiliations:** ^1^Faculty of Biomedical Sciences, Institute of Public Health, Università della Svizzera italiana, Lugano, Switzerland; ^2^Sasso Corbaro Foundation, Bellinzona, Switzerland; ^3^Ente Ospedaliero Cantonale, Area Medica Direzione Generale, Bellinzona, Switzerland

**Keywords:** COVID-19, triage, ethics, justice, autonomy, Switzerland, qualitative research

## Abstract

**Aims:** One of the major ethical challenges posed by the Covid-19 pandemic comes in the form of fair triage decisions for critically ill patients in situations where life-saving resources are limited. In Spring 2020, the Swiss Academy of Medical Sciences (SAMS) issued specific guidelines on triage for intensive-care treatment in the context of the Covid-19 pandemic. While evidence has shown that the capacities of intensive care medicine throughout Switzerland were sufficient to take care of all critically ill patients during the first wave of the outbreak, no evidence is available regarding the acceptance of these guidelines by ICU staff. The aim of this qualitative study was to explore the acceptance and perceived implementation of the SAMS guidelines among a sample of senior physicians involved in the care of Covid-19 patients in the Canton of Ticino. Specific objectives included capturing and describing physicians' attitudes toward the guidelines, any challenges experienced in their application, and any perceived factors that facilitated or would facilitate their application.

**Methods:** We conducted face-to-face and telephone interviews with a purposive sample of nine senior physicians employed as either head of unity, deputy-head of unit, or medical director in either one of the two Covid-19 hospitals in the Canton of Ticino during the peak of the outbreak. Interviews were transcribed verbatim and thematically analyzed using an inductive approach.

**Results:** We found that participants held different views regarding the nature of the guidelines, saw decisions on admission as a matter of collective responsibility, argued that decisions should be based on a medical futility principle rather than an age criterion, and found that difficulties to address end-of-life issues led to a comeback of paternalism.

**Conclusions:** Results highlight the importance of clarifying the nature of the guidelines, establishing authority, and responsibility during triaging decisions, recognizing and addressing sources of interference with patients' autonomy, and the need of a cultural shift in timely and efficiently addressing end-of-life issues.

## Introduction

One of the major ethical challenges posed by the Covid-19 pandemic comes in the form of triage decisions for critically ill patients (1). These decisions relate to the fair prioritization of patients for specific treatments (e.g., mechanical ventilation) in a situation of limited life-saving resources (2). Based on current estimates, 80% of confirmed cases of Covid-19 can be treated as outpatients, up to 20% require hospitalization, and 5% become critically ill and need intensive care (3). The Swiss Society of Intensive Care Medicine (SSICM) assessed the occupancy of bed capacities of the 82 officially recognized or certified intensive care units (ICUs) in Switzerland between March 30 and June 16, 2020 (4). The SSICM reported that, despite the sharp temporary increase in the occupancy rates during April 2020, the capacities of intensive care medicine throughout Switzerland were sufficient to take care of all critically ill patients, but found substantial regional differences, with ICUs in the Ticino and Lake Geneva regions being the busiest (4).

Medical-ethical guidelines to support decision-making in individual cases arising in the day-to-day practice of intensive-care medicine have been developed by the Swiss Academy of Medical Sciences (SAMS) in 2013 (5). In view of the extraordinary challenges that the Covid-19 pandemic has posed to the health system, and particularly to ICUs, the SAMS – in collaboration with the SSICM – supplemented the 2013 guidelines on intensive care with an annex providing precise arrangements for triage of patients in the event of a shortage of resource (6). These guidelines, which were published at the end of March 2020, mostly overlap with other triage guidelines simultaneously developed in the rest of Europe (7). They consider prognosis an indispensable precondition for maximizing benefit; refer to short-term survival only as a key triaging criterion; reject an age limit as a criterion in itself (but mention age of 85+ as an exclusion criterion to ICU admission in case of shortage of beds); cite the will of the patient as guiding treatment choices; recognize futility as a justification to end treatment even against patient will; advocate for preferential treatment for healthcare professionals (HCPs); emphasize fair decision-making processes and good palliative care; call in for interprofessional teams to make and document triage decisions fairly and transparently; demand regular re-evaluation of the decisions taken; and call for psychosocial support for HCPs (6, 7). On December 17, 2020, the guidelines were updated to reflect the most recent scientific evidence and feedback collected from various stakeholders over the previous months (6). The main changes include clarification of the meaning of the principle of short-term survival prognosis, and that it is always about making decisions that limit the number of deaths as much as possible, the importance of respecting and re-evaluating the patient's wishes (6).

Some studies suggest that, although, recommendations for ICU triage are available, compliance with them is suboptimal (8–10). Decisions on whether to accord a critically ill patient ICU admission priority in a situation of limited bed capacity are complex, and entail balancing the potential risks and benefits for the individual patient with the admission and treatment implications for future ones (11). From 30 March to April 21, 2020, a survey was conducted in Switzerland with a sample of the French- and German-speaking population to investigate the extent to which the general public agrees with the SAMS guidelines introduced in late March 2020 (12). This survey provides an overview of how these guidelines have been received by the general population. However, while evidence from southern Switzerland, which was greatly impacted during the first wave of the epidemic, is lacking, it is also unknown how physicians working on the front line at the peak of the outbreak received the SAMS guidelines, and what implementation barriers and facilitators they perceived. Qualitative research can provide valuable insights into the nature of the physicians' perception, understanding, and acceptance of the SAMS guidelines, and on how these are used and applied accounting for patients' values and preferences. Moreover, through the consideration of context, and relevant details, and the application of a recursive approach, qualitative research favors the emergence of themes and topics that can inform the design and conduction of structured investigations, including surveys aimed at describing and quantifying practices, procedures, and behaviors as the pandemic unfolds, and its transformative impact on evidence-based clinical decisions evolves unpredictably.

The aim of this qualitative study was to explore the acceptance and perceived implementation of the SAMS guidelines among a sample of senior physicians involved in the care of Covid-19 patients in the Canton of Ticino during the peak of the outbreak. Specific objectives included capturing and describing physicians' attitudes toward the guidelines, any challenges experienced in their application, and any factors that facilitated or would facilitate their application.

## Materials and Methods

### Study Design

We conducted a qualitative study employing face-to-face and telephone interviews to capture how the SAMS guidelines were received and applied by senior physicians employed in either one of the two Covid-19 hospitals in the Canton of Ticino during the peak of the pandemic. The use of the telephone as a medium for conducting the interviews was chosen to offer the greatest flexibility for the scheduling of interviews to fit in with the physicians' workload.

We recruited a sample of nine senior physicians through purposive sampling, corresponding to almost all senior physicians employed at the two hospitals (*N* = 11). To be eligible for the study, participants had to be employed as either head of unit, deputy head of unit or medical director at either the ICU, the intermediate care unit (IMCU), or emergency department (ED) of one of the two hospitals dedicated to Covid-19 patients in the Canton of Ticino during the peak of the outbreak. This allowed us to identify physicians who had gained substantial, direct experience with Covid-19 patients, and had taken a responsible role in the decision-making process regarding whether or not to accord priority to patients for intensive care. Participants were invited to the study by either e-mail or phone. All contacted participants agreed to participate.

### Data Collection

We conducted semi-structured interviews at a time convenient for participants, between April 17 and July 15, 2020. After explicit consent from participants, all interviews were audio-recorded. Based on a semi-structured guideline (Appendix 1), we asked participants open-ended questions to elicit their (1) general attitude toward the guidelines, (2) perceived general implementation of the guidelines, (3) perceived implementation of specific aspects of the guidelines (e.g., protection of the HCPs involved), (4) the decision-making processes adopted, (5) any challenges experienced in the application of the guidelines, and (6) any factors that facilitated or would facilitate their application. Interviews lasted between 28 and 56 min. The interviewer (FM) was a female researcher and social worker who, at the moment of data collection, was undertaking her postgraduate training in philosophy, and had substantial experience in qualitative research.

### Data Analysis

Audio-recordings were transcribed verbatim. One member of the research team (MF) independently conducted an inductive thematic analysis of the transcripts in the original language (Italian) following the six-stage comprehensive thematic analysis approach developed by Braun and Clarke (13). The analysis included reading the transcripts multiple times to familiarize with the text, identifying meaningful quotes regardless of their length, labeling them under broader concepts, organizing the generated labels around more general themes, and creating relationships between them. The last stage of the analysis process was devoted to identifying and highlighting thematic tensions experienced by participants. To validate the results, discussion between the interviewer and the coder took place at the end of the analysis. Disagreements in the interpretation of the findings were resolved through discussion and by making constant reference to the transcripts.

The Ethics Committee of the Canton of Ticino issued a favorable opinion on the study (Req-2020-01307). The objectives of the study and voluntary nature of participation were explained to participants both at first contact (either by phone or by e-mail) and before starting the interview (either in person or over the phone). Oral informed consent was obtained before each interview. Confidentiality was assured by replacing names with numbers and removing any identifying information from the transcripts. All audio recordings, transcripts and participants' personal data were saved on password-protected computers. In this article, we have followed the Standards for Reporting Qualitative Research guidelines (14).

## Results

### Characteristics of the Sample

The sample was composed of nine physicians, of which seven were men (Table 1). The average age was 49.4 years (SD = 8.6; range = 38–64). Five participants were employed as head of unit, one as deputy head of unit, and three as medical directors. Six participants were employed at the ICU, two at the IMCU, and one at the ED. To preserve participants' privacy and confidentiality, only participants' gender and age will be provided after each quote. We extracted four main themes from the data: (1) between a shared source of direction and an individual decision, (2) a matter of collective responsibility, (3) beyond age: a matter of futility, and (4) paternalism's comeback.

**Table 1:** Characteristics of study participants (*N* = 9).

**Variable**	**N (%)**
**Gender**	
Female	2 (22%)
**Age**	M = 49.4 (SD = 8.6; range: 38–64)
**Specialty**	
Intensive care unit	5 (55%)
Emergency	1 (11%)
Critical area	1 (11%)
Internal medicine	1 (11%)
Palliative care	1 (11%)
**Role**	
Head of unit	5 (55%)
Medical director	3 (33%)
Vice-head of unit	1 (11%)
Years of experience^*^	M = 23.5 (SD = 8.4; range: 11–39)

* *Years of experience are counted since obtaining medical degree*.

### Between a Shared Source of Direction and an Individual Decision

While almost all participants explicitly stated that they welcomed the SAMS guidelines in March 2020, they differed in the way they viewed them. On the one side, three participants viewed the guidelines as a source of direction, legitimization, and protection. As the following participant stated, the guidelines helped the team understand that they were making the right decision for the patient:

“They [the guidelines] helped us understand that we were choosing correctly.” (Participant 5, age range 51–60).

The following participant reported that the guidelines helped him because he felt that the criteria guiding his decision were broadly shared:

“Criteria help because you don't feel that the limit you set is just your decision, but rather a broadly shared directive.” (Participant 3, age range 41–50)

One participant explained that, because decisions on invasive procedures had to be made rapidly, the guidelines legitimized their decisions and ensured physicians' protection:

“We also felt entitled to make uncomfortable decisions, and especially for us in the emergency room, they were acute decisions and you had to instantly decide whether to intubate or not to intubate. We felt protected when these directives came out.” (Participant 4, age range 31–40)

On the other side, four participants stated that the guidelines should only serve as a general framework that is subject to interpretation and changes according to each physician's evaluation. According to the following participant, the attending physician should not only have the ultimate decision based on each patient's unique characteristics but also bear ultimate responsibility for any decisions:

“I think that the problem is precisely to lean on the single case and that each patient is unique and unrepeatable. Having guidelines helps and takes some of the weight off, but obviously, you have to focus on the individual case and the weight of ethical responsibility cannot be completely removed from the physician.” (Participant 6, age range 61–70)

In addition, the following participant felt legitimized in deviating from the guidelines once he understood that they were developed by a group of experts, as if this made the guidelines a “weaker” expert opinion document:

“It was of help to us to have a scheme even if, at the beginning, it was important to understand where things came from, and this was missing. I can accept anything that is written, but it must be justified. It is a group of experts, then it is an expert opinion, and this was very important to understand how far one could go from these instructions. […] When they talk about emo-dynamic instability with doses of Noradrenaline… because for us any dose of Noradrenaline is for intensive care as we don't have intermediate care, so this was changed immediately, it was very easy.” (Participant 9, age range 31–40)

One participant felt a contradiction between the intended goal of the guidelines to provide a direction and the fact that he, as a physician, has the best understanding of the patient's condition:

“On the one hand they are relieving because there is a frame of reference, on the other hand they were written for the urgency and for a disease that was not known and therefore, they are not like a cooking recipe. However, it gave us the peace of mind of having a framework to refer to. […] The guidelines give us a framework, but WE decide where to be and then apply the directives in that area, and that was the hardest thing. […] Then they are not as precise as other directives of the Swiss Academy of Medical Sciences are, and it was clear to me that they could not be. At a certain point there is a paradox, because the one who is treating the patient is me and I am the one who knows best how things are going, but they have to give me guidelines, and therefore, it is a bit contradictory. It is not a disease that you know and know what happens if you don't treat it, or what happens if you treat it. There were so many unknowns… Around me I heard people criticizing the fact that the guidelines had to be more precise, the age criterion had to be more precise… But, in the end, it was clear to me that these guidelines can only be a lighthouse that is most appropriate from an ethical and technical point of view.” (Participant 1, age range 41–50)

### A Matter of Collective Responsibility

Participants explained that the decision-making process regarding admission to ICUs in both hospitals included asking a second opinion from a senior physician operating in the other Covid-19 hospital. As the following participant reported, this process was justified because it was considered a matter of collective responsibility:

“It is a matter of collective responsibility: we organize ourselves differently if a patient needs intensive care or not and avoid unpleasant situations.” (Participant 9, age range 31–40)

In addition, as the following participant reported, asking for an external, second opinion would ensure that the responsibility would not fall on one individual only, but would be shared and documented:

“These were the admission criteria and then there are many decisions during the stay in intensive care, but even there we tried to untie the individual physician from the decision making.” (Participant 8, age range 41–50)

As the following participants explained, the decision to always include a second opinion was necessary to ensure fairness of the decision-making process:

“We set up a system whereby if you didn't want to admit a patient to the ICU, you would talk to a physician from the other institution to try to be balanced.” (Participant 1, age range 41–50)

“Such a thing would have been against the principle of justice, because by doing so we would have done something unfair to the patients who would come later.” (Participant 6, age range 61–70)

Finally, as the following participant stated, this process was also informed by a shared understanding of the short- and long-term implications of these decisions not only for patients and their families but also for the team:

“These are very difficult situations, and this is why, in our group of intensivists, we said to ourselves that we risk carrying on our shoulders these very strong decisions for a week, a month, a year… And therefore, it must not be the individual who responds. We decided internally that, if I were confronted with such a situation, I would share it with an intensivist from the other hospital. […] Therefore, with someone not directly involved with the patient's care, in order to have a shared decision and on the other hand with a certain traceability of our decision… not that one single individual decides.” (Participant 2, age range 51–60)

The same participant added that it is necessary to discuss decisions with an external physician because accepting to rely on criteria that are mandated from above can be dangerous and may threaten individual responsibility:

“We must be careful to refer to a group of decision makers because there are very dangerous psychological mechanisms, otherwise those things that happened in the Second World War will happen again… Everyone feels not responsible because they said that we must kill twenty-five Jews, so I only execute an order. In that case, I am not responsible, and I decide this way because, from above, they have decided that I will take that patient, while the other does not. We have chosen to have another intensivist referent on the same hierarchical level who does not work here.” (Participant 2, age range 51–60)

### Beyond Age: A Matter of Futility

When asked about the perceived role of patients' age as an ICU admission exclusion criterion in the decision-making process, all participants reported that age was never considered a factor *per se* and they always relied on a futility rather than a distributive justice principle. The two main reasons they cited are that age is not an absolute but a negative prognostic factor, and that they were never in stage B (Stage A: ICU beds available, but national capacity is limited, and there is reason to believe that, within a few days, ICU beds may become unavailable in Switzerland and transfers to ICUs abroad may not be possible to a sufficient extent; Stage B: No ICU beds available). As the following two participants pointed out:

“Basically, age does count because it is a negative prognostic factor, but it is not an absolute value. If possible, I would not use age as a killer factor.” (Participant 1, age range 41–50)

“Age as a single criterion has never been considered a killer criterion, luckily, since we have never been in a situation like Lombardy. We have never been in a real state of need. We went as far as to consider the criterion of futility.” (Participant 8, age range 41–50)

The core question participants asked themselves was whether admission to the ICU would meet any criteria of medical futility. One participant explained that, following a futility principle, they would ensure that decisions would not change even if resources were available:

“Even from a medical point of view, if we remove the variable concerning the availability of resources, but only look at the evolution, our suggestion would not probably change. […] Along the way, the perception changed, and we told ourselves that we had to be careful about the resources we had… But we also told ourselves that we should do neither useless things nor heroic ones, knowing that we are facing something serious.” (Participant 3, age range 41–50)

Participants cited frailty, diagnosis, and prognosis as better criteria compared to age to inform ICU admission decisions:

“If we remain bound to the numerical aspect of age, we do not get out of it. I have found that a good method is the functional reserve, that is the reserves we have. […] As the clinicians used to say in the past: such things should not be done now at this age, because they don't have the reserves, not because of age! So, we added more and more the frailty score, which is a score that geriatricians use a lot that shows you that a person who is vulnerable and dependent will never make it. It would be like asking this patient to walk to Mount Bre. This is the mechanism behind: access to intensive care is like asking the patient for something that he or she will never be able to do, so age is relativized because we look at the functional aspect.” (Participant 2, age range 51–60)

“I cannot say that, over the age of eighty, I no longer intubate anyone, but we have differentiated the two, saying that we must put the pathology and prognosis in the perspective of triage. So, these criteria are specific for Coronavirus patients because the prognosis is bad, and the intubation is long. For the others, who are usually here [in the hospital], the criteria are looser.” (Participant 3, age range 41–50)

“Age was one of the elements considered in the evaluation, but frailty and prognosis were much more important because there was a principle of non-maleficence behind it. A very frail patient would not have survived such a long stay in intensive care.” (Participant 6, age range 61–70)

Few participants mentioned that they would not feel comfortable in employing an age criterion to refuse ICU admission, but would nevertheless respect the age threshold if they entered phase B, because mortality for patients who are 80+ has been shown to be close to 100%.

“In the SAMS guidelines age was not so clear a factor, but in those of the Canton age was written and respected, because despite in some countries this limit has not been applied, mortality was practically 100%.” (Participant 5, age range 51–60)

“As a group, we agreed to limit access to intensive care above age 80 because it is known that mortality in or out of intensive care is exactly the same, so there is no gain on expectation of life and this is from an extra-Covid study that has been known for some time.” (Participant 9, age range 31–40)

In addition, one of the participants reported that the general population considers age as the main criterion to establish ICU admission priority. As the following participant reported, physicians may not use age as a criterion, but when they confront the patients' families, these will make requests based solely on such a criterion:

“Personally, I did not use age as a factor, but the family often reported age as a factor. Sometimes they said: “My mom is 90 years old, she lived her life.” There was no knowledge about the pathologies she had, but the population considers age as the real point. Others said: “He is only 70 years old.” But he had a heart disease, was cirrhotic, etc. So, there is a discrepancy there. Insiders never really considered age, but the population did.” (Participant 4, age range 31–40)

### Paternalism's Comeback

Participants reported that one of the main challenges they encountered was the difficulty to address the topic of end-of-life and of advance directives with the patients and their families. Some explained that this difficulty is due to cultural reasons:

“I cannot deny that there have been some difficulties: a cultural difficulty from Ticino, Lombardy, etc., Compared to German-speaking Switzerland, where I worked, we are much more reluctant to discuss these things here. […] Here, there is always a tendency to discuss these things at the last minute, in a very unprepared fashion, with the desire to make all family members agree… And that is something that always causes a big delay, especially in families with many children. We don't have a good culture on that. The population understands some words, like “therapeutic obstinacy,” and in fact the idea is to use these words, but not everyone is really able to understand them.” (Participant 4, age range 31–40)

One participant added that the difficulty to address end-of-life issues is also common among HCPs and not only family members:

“Colleagues lack sensitivity on this… They are unable to discuss issues of end of life, how to deal with it… And this is a constant thing.” (Participant 5, age range 51–60)

According to half of the participants, the reluctance to discuss end-of-life issues frequently led to situations in which physicians would propose a treatment pathway and families would simply accept it without questioning it. Participants referred to this phenomenon as a form of paternalism:

“Very often, patients told us to decide what we thought, so we assumed a bit of paternalism, that is, a bit of paternalism came back through the window, but in my opinion, it was not wrong. Patients were very different, and relatives too… It's a very strange thing.” (Participant 6, age range 61–70)

Most participants reported that paternalism's comeback was due to the emotional burden investing patients and family members, characterized by fear and uncertainty:

“Try to identify yourself with a son or a daughter… Doctor [X] calls home and you don't know who she is, you don't not know what role she has, you don't know if her voice really matches what she is talking about, and she says that your mother or father is very serious… Think about how difficult this is to accept.” (Participant 4, age range 31–40)

Participants also reported that the absence of the family members, who could not be close to the patients in the hospital and frequently interact with the care team, accentuated this form of paternalism and prevented a shared decision making approach.

“Who treats the patient? Physicians, nurses, and families, who are also part of the therapy. This is something that we lacked. We lacked the support of the families, the fact of having family members with the patient, who share a journey with the patient, and understand where the patient is going. They themselves told us: “We understand, we must stop, because he cannot make it.” And we missed this great help in difficult decisions. We missed one therapeutic element, which is the family.” (Participant 5, age range 51–60)

“The absence of family members, which we always asked to come when we saw that the situation was serious… They came, they stayed half an hour and left, not like the usual, when they can come and stay here. Also, the absence of patients' relatives, this loneliness, this fear, in my opinion, influenced the decisions, and I don't know how free these people were and if they were like ten days before getting sick.” (Participant 6, age range 61–70)

Participants reported that one of the main challenges they encountered was the difficulty to address the topic of end-of-life and of advance directives with the patients and their families. Some explained that this difficulty is due to cultural reasons:

“I cannot deny that there have been some difficulties: a cultural difficulty from Ticino, Lombardy, etc. Compared to German-speaking Switzerland, where I worked, we are much more reluctant to discuss these things here. […] Here, there is always a tendency to discuss these things at the last minute, in a very unprepared fashion, with the desire to make all family members agree… And that is something that always causes a big delay, especially in families with many children. We don't have a good culture on that. The population understands some words, like “therapeutic obstinacy,” and in fact the idea is to use these words, but not everyone is really able to understand them.” (Participant 4, age range 31–40)

One participant added that the difficulty to address end-of-life issues is also common among HCPs and not only family members:

“Colleagues lack sensitivity on this… They are unable to discuss issues of end of life, how to deal with it… And this is a constant thing.” (Participant 5, age range 51–60)

According to half of the participants, the reluctance to discuss end-of-life issues frequently led to situations in which physicians would propose a treatment pathway and families would simply accept it without questioning it. Participants referred to this phenomenon as a form of paternalism:

“Very often, patients told us to decide what we thought, so we assumed a bit of paternalism, that is, a bit of paternalism came back through the window, but in my opinion, it was not wrong. Patients were very different, and relatives too… It's a very strange thing.” (Participant 6, age range 61–70)

Most participants reported that paternalism's comeback was due to the emotional burden investing patients and family members, characterized by fear and uncertainty:

“Try to identify yourself with a son or a daughter… Doctor [X] calls home and you don't know who she is, you don't not know what role she has, you don't know if her voice really matches what she is talking about, and she says that your mother or father is very serious… Think about how difficult this is to accept.” (Participant 4, age range 31–40)

Participants also reported that the absence of the family members, who could not be close to the patients in the hospital and frequently interact with the care team, accentuated this form of paternalism and prevented a shared decision making approach.

“Who treats the patient? Physicians, nurses, and families, who are also part of the therapy. This is something that we lacked. We lacked the support of the families, the fact of having family members with the patient, who share a journey with the patient, and understand where the patient is going. They themselves told us: “We understand, we must stop, because he cannot make it.” And we missed this great help in difficult decisions. We missed one therapeutic element, which is the family.” (Participant 5, age range 51–60)

“The absence of family members, which we always asked to come when we saw that the situation was serious… They came, they stayed half an hour and left, not like the usual, when they can come and stay here. Also, the absence of patients' relatives, this loneliness, this fear, in my opinion, influenced the decisions, and I don't know how free these people were and if they were like ten days before getting sick.” (Participant 6, age range 61–70)

## Discussion

Allocation of scarce life-saving interventions in accordance with generally accepted ethical principles is a major challenge of the current pandemic and guidelines are available in many countries to provide support for rationing decisions. We aimed to explore how senior physicians involved in the care of Covid-19 patients during the first wave of the pandemic accepted and implemented locally issued guidelines on triage for ICU admission. We found that participants held different views regarding the nature of the guidelines, saw decisions on admission as a matter of collective responsibility, argued that decisions should be based on a medical futility principle rather than an age criterion, and found that difficulties to address end-of-life decisions led to a comeback of paternalism. In the next paragraphs, we contextualize our findings, and interpret their implications accounting for the limitations of the study.

Our finding that some participants viewed the guidelines as a source of protection resonate with the need for hospital leadership to ensure legal safeguard prior to establishing a triage system in order to ensure consistent application of triage protocols (15). In line with other studies conducted in Europe (10), half of our participants were aware of the guidelines but stated that they would adapt them according to their personal expertise and preferences. This is consistent with the argument that triage algorithms and protocols can be useful but can never replace the role of trained intensivists building their decisions on the involvement of multidisciplinary teams (16), and should therefore provide a general framework to be adapted to local health systems (17). However, such a variation in the application of national triage protocols is problematic and might represent a potential source of discrimination, as criteria for exclusion are selectively applied to only some types of patients, rather than to all patients being considered for critical care (18). Indeed, studies showed that reasons for poor compliance with ICU triage guidelines were unfamiliarity with the guidelines and disagreement with the fundamental approach underlying the guidelines (19).

In line with previous evidence, we found that collaborative decision-making facilitated choices on ICU admission (20). Our participants reported having involved external, senior physicians in the decision-making process on whether to accord priority to patients for intensive care. This can be due to awareness of the psychological implications of making ICU admission decisions. Such decisions have been previously described as being extremely difficult and emotionally burdensome, as physicians feel they are making life-death decisions (20–22). Several guidelines have recommended implementing specific programs to enhance HCPs' resilience to cope with the psychological burden triggered by this pandemic (15), and the SAMS state that HCPs are to be protected as far as possible against excessive psychological stress (6). The strategy to discuss ICU triage decisions with external physicians may also be due to participants' awareness that personal attitude may be a key driver of the decision and could jeopardize the fair allocation of limited resources. Variation in intensive care unit admission decision-making due to personal attitudes has been previously found by previous studies (23, 24). Extending responsibility for triage decisions to external decision-makers may also be due to a current controversy on who should have the authority to make such choices, and how physicians should best be supported (25). This is in line with recommendations from a task force of the World Federation of Societies of Intensive and Critical Care Medicine that triage should be led by intensivists considering input from nurses, emergency medicine professionals, hospitalists, surgeons, and allied professionals (16). Previous qualitative studies conducted in lower-middle income countries found that communication between staff constituted an obstacle to good quality care and identification of the critically ill patients (9). In contrast, our participants cited discussion with external physicians as a key facilitator of the decision-making process.

Participants also shared the view that age should not be a criterion for limiting intensive care. This reflects the principle that scarce resources should be fairly allocated regardless of age, sex or gender identity, race or ethnicity, religion, socioeconomic status, and similar individual factors (17, 26, 27), and that priority decisions should be primarily based on medical criteria (19, 28). However, frameworks have been proposed that give individuals who perform tasks vital to the public health response enhanced priority (29). Moreover, ethical dilemmas regarding rationing, allocation, and prioritization are not only a consequence of the severity of the Covid-19 disease and scarcity of life-saving resources, but also of how the concept of justice and other values are interpreted by care teams (30). Our study participants reported to be committed to the rule of rescue over the good of the many (31). This is consistent with the findings of a recent review of the literature which found that age 85+ is one of the least ranked criterion for exclusion (32), but not in line with previous evidence that patient's age had the largest impact at ICU admission (33). Beyond evidence, age represents an area of disagreement also among international triage recommendations (34). Next, criteria to withdrawing life support from one patient to provide it to another were not cited by our participants as reasons of contention, but the literature suggests that more evidence and guidance are needed (18). A recent review of more than one hundred research articles, guidelines and reviews on the topic of ICU resource allocation found that patient preference was the most common reason cited to exclude patients from ICU admission (32).

Interestingly, our participants referred to a revival of the paternalistic model, because they argued that physicians could not always carefully ascertain the patients' and their families' will, due to logistic and emotional barriers, many of which were unprecedented and conceivably exceptional during the first wave of the epidemic in Southern Switzerland. And yet, previous evidence has shown that end-of-life decisions were perceived as more complex in the absence of family or of information about patients' end-of-life preferences, and when there was time pressure and a lack of training in end-of-life decision-making (21, 35, 36). Studies also found that patients with active advance directives were less likely to be admitted to the ICU (11, 37, 38). To give precedence to respect of a distributive justice principle, our participants reported to have downgraded principles of autonomy and beneficence (39, 40). This is in line with larger quantitative studies which found that patient-related factors were rated higher on their potential to affect decisions than scarcity-related or administrative-related factors (41). Our participants' difficulty in addressing end-of-life issues with patients and their families stresses the importance of providing just-in-time training and simulation sessions for non-ICU clinicians reassigned to work in ICU, to better prepare them for their roles and for addressing sensitive matters (15). Previous studies have identified specific cultural beliefs, values, and communication patterns that can be used to promote cultural competency among practitioners who provide care at end of life (42).

Our results have several potential implications. Since our participants reported to view the guidelines as either an inalterable set of instructions or a general framework apt to changes, national triage guidelines should clarify to what extent protocols can be adapted, which is key for acceptance, adherence, and integration in clinical practice. Furthermore, our results suggest that participants implemented a shared strategy to manage the individual responsibility of making ICU admission decisions (i.e., involving an external, senior physician in the decision-making process). This finding stresses the importance of clarifying issues of authority and responsibility during triaging decisions. A possible solution could be to establish functional roles and responsibilities of the internal personnel and interface agencies or sectors at national, regional, local, facility, and hospital levels, while providing appropriate training of triage staff (43). In addition, we found a shared belief that the age criterion should not be used as a criterion *per se* but as a prognostic factor. Guidelines should better explain the role of such criterion in guiding ICU admission and stay decisions, and provide supporting evidence from the literature. Finally, as a number of barriers make it difficult to ascertain the patient's wishes with regard to emergency treatment and intensive care at an early stage, training should be offered to healthcare staff to address end-of-life issues while campaigns and other activities should be promoted to raise public awareness of the importance of discussing and drafting one's advance directives.

## Limitations and Conclusions

Some limitations of our study are worth noting. First, this is a qualitative study conducted in the Italian-speaking Canton of Switzerland with a small sample of participants. While a substantial portion of the senior staff involved in the care of Covid-19 patients during the first wave of the pandemic was included in the study, our results should be generalized to other geographical and cultural contexts with caution. Second, we cannot exclude that participants answered our questions in a way that would be seen socially accepted. To mitigate such possible social desirability bias, we reassured participants that all the information they would share would be kept confidential. Third, the format of the interviews was semi-structured to limit its duration and fit in with the physicians' workload. While this allowed us to maximize our sample and answer our research question in a targeted way, opting for an in-depth format might have led to different results. Fourth, our interviews focused on the first wave of the pandemic, when participants did not have to face decisions on withdrawing intensive care treatment (the “bad” period) (44). Conducting the study during a period of more intense patient influx and higher demand of ICU beds (the “ugly” period) may have led to different results (44), but health services were, nonetheless, functioning close to their maximum capacity.

As cases increase exponentially, the need to optimize rationing decisions and triage of patients with Covid-19 at all health facility levels will intensify. Evidence-based guidelines should seek to address all the questions related to ICU admission, discharge, and triage, including questions regarding the healthcare team's perceived acceptance of the guidelines, and any barriers and facilitators to their implementation (45). Our results stress the importance of sensitizing both healthcare professionals and the general population regarding the intended nature of the guidelines, the benefits of discussing and compiling one's advance directives, and including family members as much as possible in the decision-making process regarding patients' ICU admission and stay. We call for policy makers to intensively engage with diverse groups (citizens, HCPs, ethicists, and disaster medicine experts) in the refinement of the guidelines and for future research to investigate the acceptance of the SAMS guidelines in other Swiss Cantons.

## Data Availability Statement

The datasets presented in this article are not readily available because there are ethical restrictions on sharing a de-identified data set as (1) data contain potentially sensitive information and (2) participants did not provide explicit consent to share indirectly identifiable data. For these reasons, we can only share portions of the de-identified data set. Requests to access the datasets should be directed to Ethics Committee of the Canton of Ticino, tel.: +41 91 814 30 57, e-mail: dss-ce@ti.ch.

## Ethics Statement

The study was reviewed and authorized by the Ethics Committee of the Canton of Ticino. The participants provided their written informed consent to participate in this study.

## Author Contributions

RM and ML made all the contacts with the participants. FM conducted the interviews. MF and FM led the analytic process and analyzed the results. MF, FM, and RM contributed to the study design. MF verified the findings of the analysis and wrote the paper. All authors contributed to reviewing the paper.

## Conflict of Interest

The authors declare that the research was conducted in the absence of any commercial or financial relationships that could be construed as a potential conflict of interest.

## Publisher's Note

All claims expressed in this article are solely those of the authors and do not necessarily represent those of their affiliated organizations, or those of the publisher, the editors and the reviewers. Any product that may be evaluated in this article, or claim that may be made by its manufacturer, is not guaranteed or endorsed by the publisher.
