# Supplementary material for: Physicians' Acceptance of Triage Guidelines in the Context of the COVID-19 Pandemic: A Qualitative Study
Source: Front Public Health. 2021 Jul 30;9:695231. doi: 10.3389/fpubh.2021.695231 (PMC8360847; doi:10.3389/fpubh.2021.695231)
Supplement: Supplementary file 1 [file Table_1.DOCX]

**Appendix 1. Interview grid**

| Ice-breaking question | How would you describe the situation in your unit, now that the peak is over? |
| --- | --- |
| General attitude towards the guidelines | Have you heard of the SAMS guidelines?  What is your general opinion on these guidelines?  How would you evaluate these guidelines in terms of clarity?  How would you evaluate these guidelines in terms of precision? |
| Perceived general implementation of the guidelines | To what extent were these guidelines followed in your unit?  To what extent did these guidelines align with previous practice?  To what extent were the guidelines compatible with the facility? |
| Perceived implementation of specific aspects: *Protection of the professionals involved* | In which ways were the HCPs protected in your unit? Think about both the physical and mental health. |
| Perceived implementation of specific aspects: *Importance of discussing in advance the patients’ wishes.* | When do you think that patients’ wishes should be clarified?  What role should the patient's family/friends play in this process?  Have you noticed an increase in advance directives during this period?  Have you ever felt that the patient's wishes did not correspond with the proposed or adopted therapeutic decision? What happened? |
| Perceived implementation of specific aspects: *Age* | What was the role of age in the decision-making process regarding admission to the ICU? |
| Decision-making process | Can you describe the decision-making process regarding decisions on procedures and therapies? Who took part in this process? |
| Barriers to the implementation of the guidelines | How difficult was to adhere to these guidelines?  What made it difficult? |
| Facilitators to the implementation of the guidelines | What did you need to follow these guidelines?  What specific skills were necessary to apply these guidelines?  What could have been done to follow them better? |
| Final remarks | Is there anything that you would like to add? |
